# The Relationships of Deteriorating Depression and Anxiety With Longitudinal Behavioral Changes in Google and YouTube Use During COVID-19: Observational Study

**DOI:** 10.2196/24012

**Published:** 2020-11-23

**Authors:** Boyu Zhang, Anis Zaman, Vincent Silenzio, Henry Kautz, Ehsan Hoque

**Affiliations:** 1 Department of Computer Science University of Rochester Rochester, NY United States; 2 Department of Urban-Global Public Health Rutgers University Piscataway and Newark, NJ United States

**Keywords:** mental health, anxiety, depression, Google Search, YouTube, pandemic, COVID-19

## Abstract

**Background:**

Depression and anxiety disorders among the global population have worsened during the COVID-19 pandemic. Yet, current methods for screening these two issues rely on in-person interviews, which can be expensive, time-consuming, and blocked by social stigma and quarantines. Meanwhile, how individuals engage with online platforms such as Google Search and YouTube has undergone drastic shifts due to COVID-19 and subsequent lockdowns. Such ubiquitous daily behaviors on online platforms have the potential to capture and correlate with clinically alarming deteriorations in depression and anxiety profiles of users in a noninvasive manner.

**Objective:**

The goal of this study is to examine, among college students in the United States, the relationships of deteriorating depression and anxiety conditions with the changes in user behaviors when engaging with Google Search and YouTube during COVID-19.

**Methods:**

This study recruited a cohort of undergraduate students (N=49) from a US college campus during January 2020 (prior to the pandemic) and measured the anxiety and depression levels of each participant. The anxiety level was assessed via the General Anxiety Disorder-7 (GAD-7). The depression level was assessed via the Patient Health Questionnaire-9 (PHQ-9). This study followed up with the same cohort during May 2020 (during the pandemic), and the anxiety and depression levels were assessed again. The longitudinal Google Search and YouTube history data of all participants were anonymized and collected. From individual-level Google Search and YouTube histories, we developed 5 features that can quantify shifts in online behaviors during the pandemic. We then assessed the correlations of deteriorating depression and anxiety profiles with each of these features. We finally demonstrated the feasibility of using the proposed features to build predictive machine learning models.

**Results:**

Of the 49 participants, 49% (n=24) of them reported an increase in the PHQ-9 depression scores; 53% (n=26) of them reported an increase in the GAD-7 anxiety scores. The results showed that a number of online behavior features were significantly correlated with deteriorations in the PHQ-9 scores (*r* ranging between –0.37 and 0.75, all *P* values less than or equal to .03) and the GAD-7 scores (*r* ranging between –0.47 and 0.74, all *P* values less than or equal to .03). Simple machine learning models were shown to be useful in predicting the change in anxiety and depression scores (mean squared error ranging between 2.37 and 4.22, *R*^2^ ranging between 0.68 and 0.84) with the proposed features.

**Conclusions:**

The results suggested that deteriorating depression and anxiety conditions have strong correlations with behavioral changes in Google Search and YouTube use during the COVID-19 pandemic. Though further studies are required, our results demonstrate the feasibility of using pervasive online data to establish noninvasive surveillance systems for mental health conditions that bypasses many disadvantages of existing screening methods.

## Introduction

### Background

Worldwide mental health problems such as depression, anxiety, and suicidal ideation have severely worsened during the COVID-19 pandemic [1-3], specifically for college students [4-7]. Yet, current methods for screening mental health issues and identifying vulnerable individuals rely on in-person interviews. Such assessments can be expensive, time-consuming, and blocked by social stigma, not to mention the reluctancy induced by travel restrictions and exposure risks. It has been reported that few patients in need were correctly identified and received proper mental health treatments on time under the current health care system [8,9]. Even with emerging telehealth technologies and online surveys, the screening requires patients to actively reach out to care providers.

At the same time, because of the lockdown caused by the global pandemic outbreak, people’s engagements with online platforms underwent notable changes, particularly in search engine trends [10-12], exposures to media reports [13,14], and through quotidian smartphone use for COVID-19 information [5]. Reliance on the internet has significantly increased due to the overnight change in lifestyles, for example, remote working and learning, imposed by the pandemic on society. The sort of content consumed, the time and duration spent online, and the purpose of online engagements may be influenced by COVID-19. Furthermore, the digital footprints left by online interactions may reveal information about these changes in user behaviors.

Most importantly, such ubiquitous online footprints may provide useful signals of deteriorating mental health profiles (eg, depression and anxiety) of users during COVID-19. They may capture insights into what was going on in the mind of the user through a noninvasive manner, especially since Google and YouTube searches are short and succinct, and can be quite rich in providing the real-time cognitive state of a person. On one hand, online engagements can cause fluctuations in mental health. On the other hand, having certain mental health conditions can cause certain types of online behaviors. This opens up possibilities for potential health care frameworks that leverage pervasive computing approaches to monitor mental health conditions and deliver interventions on time. However, the findings of this study do not imply any causal relationship between specific types of online activities and one’s level of anxiety or depression at a given point in time.

### Prior Work

Extensive research has been conducted on a population level, correlating mental health problems with user behaviors on social platforms [15,16], especially among young adolescents. Researchers monitored Twitter to understand mental health profiles of the general population, such as suicidal ideation [17] and depression [18]. Similar research has been done with Reddit, where anxiety [19], suicidal ideation [17], and other general disorders were studied [20,21]. Another popular public platform is Facebook, and experiments have been done studying anxiety, depression, body-shaming, and stress online [22,23]. In addition, it has been shown that college student communities rely heavily on YouTube for both academic and entertainment purposes [24,25]. Yet, abundant use may lead to compulsive YouTube engagements [26], and researchers have found that social anxiety is associated with YouTube consumption in a complex way [27].

During COVID-19, multiple studies have reported deteriorating mental health conditions in various communities [1-3,28] such as nationwise [29,30], across the health care industry [31,32], and among existing mental health patients [33]. Recently, it has been shown that greater use of social media during COVID-19 may induce increasing levels of anxiety and depression at both population and individual levels [14,34]. In addition, online behaviors during COVID-19 have been explored, especially for web searches related to the pandemic [10-12] and abnormal TV consumption during the lockdown [13]. Many of the behavioral studies also discussed the effects of online interactions on the spread, misinformation, knowledge, and protective measures of COVID-19, including the roles of YouTube [35-37] and other platforms [38]. Lyu et al [39] investigated hate speech targeting the Chinese and Asian communities on Twitter during COVID-19. A study in 2009 showed the opposite effect in mental health risk factors: a communitywide crisis may reduce self-harm ideation behaviors [40].

Ubiquitous data has been proved to be useful in detecting mental health conditions. Mobile sensor data such as GPS logs [41,42]; electrodermal activity; and sleep behavior, motion, and phone use patterns [43,44] have been applied in investigating depressive symptoms. Zaman et al [45] found that individual private Google Search histories can be used to detect low self-esteem conditions among college students. Huckins et al [5] examined the longitudinal changes in mental health and smartphone use through ecological momentary assessments during COVID-19 among college populations. Although studies exploring anxiety and depression have been conducted in the past, none of them have leveraged individual-level Google Search and YouTube activity logs to examine the effect of COVID-19 on college students.

### Goal of This Study

It has been shown that online platforms preserve useful information about the mental health conditions of users, and COVID-19 is jeopardizing the mental well-being of the global community. Thus, we demonstrate the richness of online engagement logs and how they can be leveraged to uncover alarming mental health conditions during COVID-19. In this study, we aim to examine whether the changes in user behaviors during COVID-19 have a relationship with deteriorating depression and anxiety profiles. We focused on Google Search and YouTube use, and we investigated if the behavior shifts when engaging with these two platforms signify worsened mental health conditions.

The scope of the study covers undergraduate students in the United States. We envision this project as a pilot study; it may lay a foundation for mental health surveillance and help delivery frameworks based on pervasive computing and ubiquitous online data. Compared to traditional interviews and surveys, such a noninvasive system may be cheaper and efficient, and avoid being blocked by social stigma while notifying caregivers on time about individuals at risk.

## Methods

### Recruitment and Study Design

**Figure 1:**
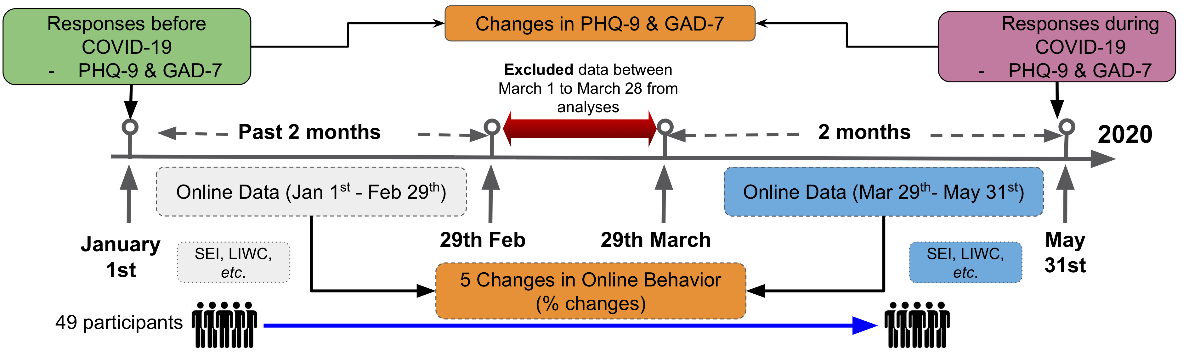
The study recruitment procedure and feature development process. All of the participants moved to remote learning on March 7, 2020, the same day a state of emergency was declared in New York State. To avoid any acute behavior during the transition to remote learning, we excluded the data from March 1 to 28. GAD-7: General Anxiety Disorder-7; LWIC: Linguistic Inquiry and Word Count; PHQ-9: Patient Health Questionnaire-9; SEI: short event interval.

We recruited a cohort of undergraduate students, all of whom were at least 18 years of age and have an active Google account for at least 2 years, from the University of Rochester River Campus, Rochester, NY. Participation was voluntary, and individuals had the option to opt out of the study at any time, although we did not encounter any such cases. We collected individual-level longitudinal online data (Google Search and YouTube) in the form of private history logs from the participants. For every participant, we measured the depression and anxiety levels via the clinically validated Patient Health Questionnaire-9 (PHQ-9) and General Anxiety Disorder-7 (GAD-7), respectively. Basic demographic information was also recorded. There were in total two rounds of data collection: the first round during January 2020 (prior to the pandemic) and the second round during May 2020 (during the pandemic). During each round and for each participant, the anxiety and depression scores were assessed, and the change in mental health conditions was calculated in the end. The entire individual online history data up until the date of participation was also collected in both rounds from the participants. Figure 1 gives an illustration of the recruitment timeline and two rounds of data collections. All individuals participated in both rounds and were compensated with US $10 Amazon gift cards during each round of participation.

Given the sensitivity and proprietary nature of private Google Search and YouTube histories, we leveraged the Google Takeout web interface [46] to share the data with the research team. Prior to any data cleaning and analysis, all sensitive information such as the name, email, phone number, social security number, and credit card information was automatically removed via the Data Loss Prevention application programming interface (API) [47] of Google Cloud. For online data and survey response storage, we used a Health Insurance Portability and Accountability Act–compliant cloud-based secure storing pipeline. The whole study design, pipelines, and survey measurements involved were similar to our previous setup [45] and have been approved by the Institutional Review Board of the University of Rochester.

To address participation bias, the study was advertised among the college population via campus wide digital announcements. The text in the study advertisements and consent materials were generic with text such as “help uncover mental health understanding via your online activities.” There was no explicit mention of anxiety or depression in the advertisement. Participation was voluntary with the option to opt out of the study anytime, and their data would not be part of the research study. The intent of the study was clearly explained at the beginning of the recruitment process via one-on-one interviews with the recruiter. We did not have anyone declining to participate or withdrawing in the middle of the study.

### Online Data Processing and Feature Extractions

The Google Takeout platform enables users to share the entire private history logs associated with their Google accounts, and as long as the account of the user was logged-in, all histories would be recorded regardless of which device the individual was using. Each activity in Google Search and YouTube engagement logs were time stamped, signifying when the activity happened to the precision of seconds. Furthermore, for each Google Search, the history log contained the query text input by the user. It also recorded the URL if the user directly inputted a website address to the search engine. For each YouTube video watched by the user, the history log contained the URL to the video. If the individual directly searched with one or more keywords on the YouTube platform, the history log also recorded the URL to the search results.

To capture the change in online behaviors for the participants, we first introduced a set of features that quantified certain aspects of how individuals interact with Google Search and YouTube. The set of features was calculated for each participant separately. Individual-level behavior changes were then obtained by examining the variations of the feature between January to March 1, 2020 (a week before the state of emergency in New York State), and March 28 to May 2020 (after the outbreak, following the lockdown and mandated social distancing).

We excluded the online data generated between March 1, 2020, and March 28, 2020, to account for any acute or temporal behavior changes concentrated around the initial lockdown or due to adapting to remote work. We focused on the persistent and stabilized online behaviors throughout the time after the lockdown. Furthermore, the spring break at our institution started on March 7, and the state of emergency in New York State was issued on the same day. All students were asked to leave campus at the start of spring break and complete the rest of the semester remotely.

Concretely, we defined 5 features and cut the longitudinal data of each participant into two segments: (1) from January 1 to February 29, 2020, and (2) from March 29 to May 31. Each segment spanned 2 months. We excluded online data from March 1 to March 28 to account for the fact that not all individuals transitioned into work from home on a specific date or practiced a strict social distancing lifestyle, although all of our participants are residents of New York State. The same feature was extracted from both segments of data, and the change was calculated. Such change was referred to as the behavior shifts during the pandemic and lockdown. Figure 1 gives an illustration of data segmentations and feature development pipelines.

#### Online Activity Distributions

We considered, for each participant, how the Google Search and YouTube activities were distributed across the 24 hours of a day before and after the lockdown, given the previously defined dates. For each trimmed data segment, we cumulated the total number of activities, regardless of Google Search or YouTube, that happened in each of the 24 hours. Thus, we obtained two 24-bin histograms, representing the activity distributions before (D_before_) and after (D_after_) the lockdown.

**Figure 2:**
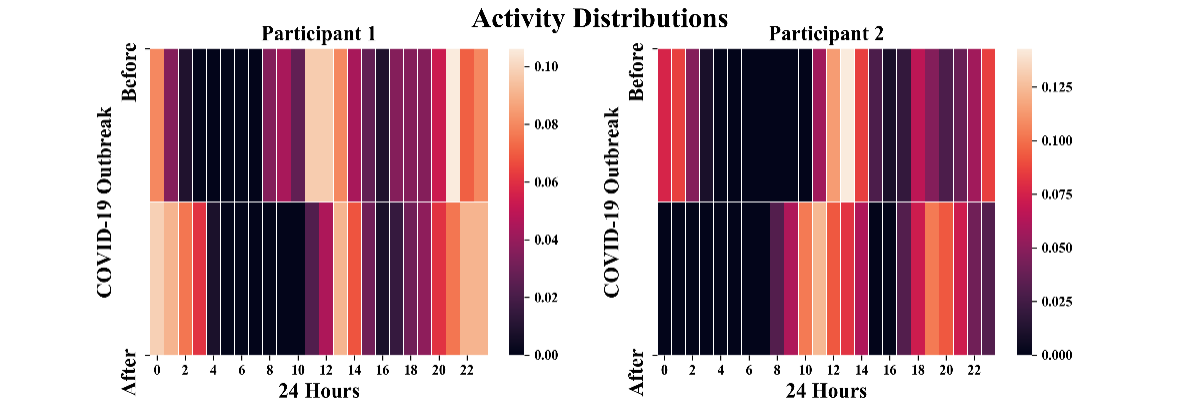
The normalized activity distributions over 24 hours before and after the outbreak of COVID-19 for two example participants.

Figure 2 showcases the normalized distributions before and after the outbreak for two participants, each cumulates 2 months of data. For participant one (PHQ-9 increased by 8 and GAD-7 increased by 3) before the outbreak, a few activities started to appear at 8 AM. After the outbreak, these early morning activities disappeared. In addition, a considerable amount of online activities appeared during late-night hours. These patterns most likely indicated a delay in bedtime. For participant two (PHQ-9 decreased by 2 and GAD-7 decreased by 6), there were several activities during late night hours before the lockdown. Followed by a long absence from Google Search and YouTube, the next event usually appeared around noon. After the lockdown, the first activity of the day started to appear in the early morning, and those late-night activities disappeared. Similarly, participant two may also have had afternoon classes at around 3 PM-4 PM. Notice that these two random cases were chosen simply to represent the fact that study participants reacted nonuniformly to the lockdown.

After that, for each of the 24 hours (*h*) of a user, we calculated the percentage (ie, relative) change of online activities before and after the lockdown:


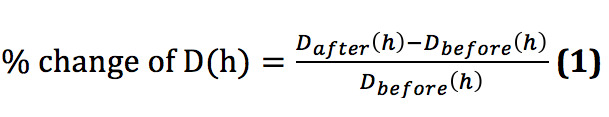


For the rest of the study, any mentioned percentage or relative changes of features were calculated in this way.

#### Last Seen Activities

**Figure 3:**
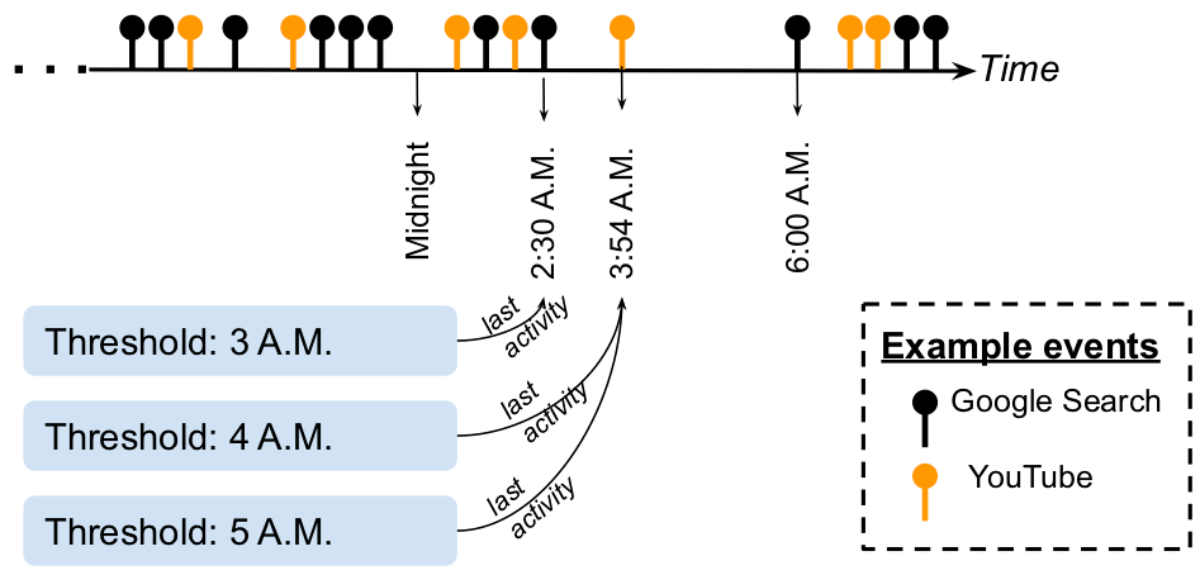
An example to demonstrate how the last seen activities are selected for different threshold hours.

We further considered the last seen activity, regardless of Google Search or YouTube, of each user in a day. It is reasonable to assume that, given the nature of our college student population, the last event before they go to bed does not necessarily happen before midnight. Strictly speaking, our goal was to capture the last event before they went to bed. Therefore, we set a threshold at late night or early morning and considered the last online activity before it. Since a discrete threshold was used, we tried several cutoff hours to perform sensitivity analyses. For our study population, we observed that the hourly volume of Google Search and YouTube activities started to decrease after midnight, and it reached the minimum at 5 AM. This pattern was periodic and persistent across our longitudinal data. Motivated by this observation, we tried a cutoff hour of midnight, 1 AM, 2 AM, 3 AM, 4 AM, and 5 AM, and counted the last events before these thresholds for each participant. Different from the aforementioned *online activity distributions*, which measures the volume of activities on Google Search and YouTube hourly, the last seen events focused solely on participants staying up late. An example illustration of the *last seen activities* is provided in Figure 3.

With each threshold, we obtained two distributions of the last seen event time stamps before and after the lockdown from each participant. On a continuous scale, we then picked the two medians of the last seen event time stamps before and after the lockdown and took the difference. For example, a difference of 1.5 hours means that the median time of last seen events shifted 1.5 hours later after the lockdown. A difference of –0.3 hours means the median time of last seen events shifted 0.3 hours earlier after the lockdown. All the time differences are in the unit of hours. There is no need to distinguish between Google Search or YouTube for this feature as we are merely looking for the last event, which could be either.

#### Short Event Intervals

We defined a short event interval (SEI) as the period of time that is less than a certain threshold (eg, 5 minutes) between two adjacent events. It usually occurs when one is consuming several related YouTube videos or is searching for similar content. Taking into consideration that YouTube and Google Search may have different thresholds to define a user session, we adapted the method in Halfaker et al’s [48] study to identify proper thresholds for consecutive activities on each of the platforms. After obtaining the session thresholds through mixture models, we counted the total numbers of such SEIs for each participant before (*SEI_before_*) and after (*SEI_after_*) the outbreak. We calculated the relative change of SEI the same way as in Equation 1 and used it as a behavioral feature.

#### Linguistic Inquiry and Word Count Attributes

The Linguistic Inquiry and Word Count (LIWC) is a toolkit used to analyze various emotions, cognitive processes, social concerns, and psychological dimensions in a given text by counting the numbers of specific words [49]. It has been widely applied in research involving social media and mental health. For the complete list of linguistic and psychological dimensions LIWC measures, see [49]. We segmented the data log for each participant by the previously mentioned dates as two blobs of texts and analyzed the words using LIWC.

Since the contexts and linguistic properties of Google Search and YouTube may be distinct, we extracted the LIWC features from them separately. For Google Search, we inputted the raw query text; for YouTube, we inputted the video title and the YouTube query text, if any. There were in total 51 different LIWC attributes. LIWC outputted the count of words falling in each dimension among the whole text. We quantified the shift in behavior by calculating the percentage change of words in each dimension after the outbreak.

#### Google Search and YouTube Categories

We labeled each Google Search query with a category using the Google natural language processing (NLP) API [50]. We used the official YouTube API to retrieve the information of videos watched by the participants, including the title, duration, number of likes and dislikes, and default YouTube category tags. For a comprehensive list of Google NLP category labels and default YouTube category tags, please refer to [51,52]. There were several categories overlapping with the LIWC dimensions, such as *health* and *finance*, and we regarded the LIWC dimensions as a more well-studied standard. Instead, we focused on the number of activities belonging to the *adult* (specifically originating from Google Search logs) and *news* categories, which were not presented in the LIWC.

Concretely, activities such as visiting a porn site (identified via the URL) and searching explicitly for information related to porn and mature content were labelled as *adult*. There was no other ambiguous nonpornographic material being categorized as *adult*. We used Google Cloud Content Classification API for labeling the search queries and used the Webshrinker [53] API to categorize the domain of every URL an individual visited. We calculated the relative changes of activities in these two categories as the behavior shifts for each participant (the same as Equation 1).

We now present a qualitative example of behavior changes in Google Search and YouTube categories. Textbox 1 showcases, for a single example participant, the top five Google Search and YouTube categories before and after the lockdown, defined by the percentages out of the total activity volume. For Google Search, we observed the disappearances of *food and drinks* (including searching for restaurants) and *shopping* from the list. In contrast, the numbers of searches related to the *beauty and fitness*, *home* (including kitchen and cooking subcategories), and *health* topics increased during the quarantine. The *reference* category was largely composed of academic content such as dictionaries, humanity and history references, and scientific proceedings. For YouTube, videos belonging to the *education* category boosted during the remote learning period after the lockdown, as did *film and animation*. The *travel and events*, and *sports* topics vanished from the list. Note that this was merely a single example, and the traits reflected here may be personal, uncorrelated to anxiety or depression, or prevalent among everyone.

**The top five Google Search and YouTube categories for an example participant before and after the lockdown.**

**Top five Google Search categories**

- Before lockdown
  Art and entertainment
  Reference
  Food and drinks
  Shopping
  Beauty and fitness
  Finance
- After lockdown
  Art and entertainment
  Reference
  Beauty and fitness
  Home
  Health
  Food and drinks

**Top five YouTube video categories**

- Before lockdown
  Music
  Travel and events
  Sports
  News and politics
  Education
  Film and animation
- After lockdown
  Music
  Education
  Film and animation
  News and politics
  Pets and animals
  Comedy

### Measurement Outcomes

#### Measurements for Changes in Online Behaviors

There were in total 5 scalar continuous dependent variables measuring various aspects of the changes in online behavior for each participant, as previously defined. These variables were extracted from two segments of the online data logs, namely, the data before and after the pandemic outbreak. All of the measurements were in percentage changes. For the *online activity distributions*, there were 24 measurements for each hour of a day. For the *last seen events*, there were several thresholds for sensitivity analyses. For the *SEIs*, Google Search and YouTube activities were considered separately with their own fitted session intervals.

#### Measurements for Mental Health Conditions

For both rounds of the data collection, anxiety levels were assessed using the GAD-7 survey, and depression levels were assessed using the PHQ-9 survey. With two rounds of surveys reported before and after the outbreak, the change in mental health conditions of each participant was obtained. According to Spitzer et al [54] and Rutter and Brown [55], an increase greater than or equal to 5 in the GAD-7 score may be clinically alarming. Similarly, as stated by Kroenke [56], an increase greater than or equal to 5 in the PHQ-9 score may indicate the need for medical interventions.

#### Demographics

In addition to the online data and mental health surveys, we also collected basic demographic information such as school year, gender, and nationality.

### Statistical Analysis

Before any analysis of mental health conditions, to eliminate the possibility of annual confounding factors interfering with the shifts in online behaviors, two-tailed paired independent *t* tests were performed. We inspected, in terms of the five quantitative features, whether the online behavior changes happened every year, such as due to seasonal factors, or only during COVID-19 for the whole study population. As previously mentioned, we collected the entire Google history log back to the registration date of the Google accounts of all participants.

We now use the example of *SEIs* to illustrate the idea. For each participant, we obtained 4 *SEIs* counts from 4 periods of time: January 1, 2019, to February 28, 2019; March 29, 2019, to May 31, 2019; January 1, 2020, to February 29, 2020; and March 29, 2020, to May 31, 2020. These counts are represented as 4 points on a Cartesian coordinate plane, where the y-axis represents the counts and the x-axis represents the time. We then calculated the slope (*S*) of the line connecting the two points from the same year. With the aforementioned process, we achieved two measurements for each participant, namely, *S*_2019_ and *S*_2020_. Viewing all the participants as a cohort, we computed the *S*_2019_ and *S*_2020_ for all features and performed multiple paired *t* tests. This enabled us to estimate the seasonal confounding factors. We could not perform the paired *t* tests on the changes of behavioral features directly because it may only validate a change in the intercept (baseline) amount of activity while ignoring the slope for each feature.

In the main experiments, with each of the aforementioned 5 features and various thresholds, we investigated the correlation of online behavioral changes with deteriorations in the GAD-7 and PHQ-9 scores, which did not require arbitrary discretization decisions. The dependent variables were the 5 behavior changes extracted from the longitudinal individual online data. Experiments were carried out in a one-on-one fashion: anxiety or depression condition was the single independent variable, and one of the five online behavior changes was the single dependent variable each time. Both of them were continuous variables.

## Results

### Study Population Statistics

We recruited 49 participants in total, and all of them participated in both rounds of the study (100% response rate). On average, each participant made 2357 (95% CI 2106.28 to 2433.45) Google Searches and 2901 (95% CI 2556.92 to 3248.67) YouTube interactions from January to February 29, 2020, and 2497 (95% CI 2069.45 to 2901.34) Google Searches and 3105 (95% CI 2702.48 to 3487.56) YouTube interactions from March 29 to the end of May. Of the 49 participants, 49% (n=24) of them reported an increase in the PHQ-9 score, and 53% (n=26) of them reported an increase in the GAD-7 score. An increase in the PHQ-9 score≥5 was reported by 41% (n=20) of participants, and 45% (n=22) of them reported an increase in the GAD-7 score≥5.

**Figure 4:**
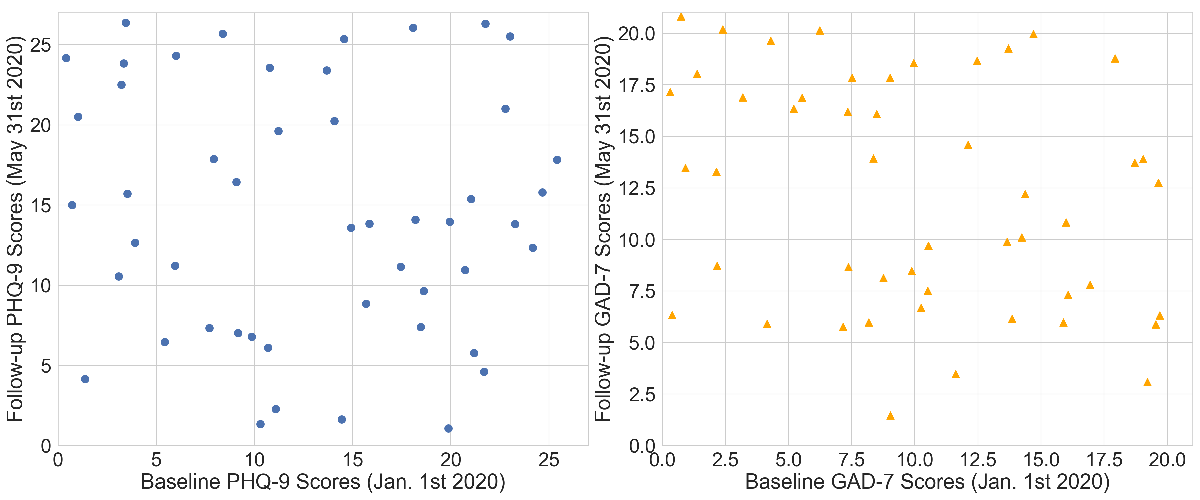
The distributions of PHQ-9 depression and GAD-7 anxiety scores before and after the lockdown. The PHQ-9 scores are shown on the left, and the GAD-7 scores are shown on the right. GAD-7: General Anxiety Disorder-7; PHQ-9: Patient Health Questionnaire-9.

**Figure 5:**
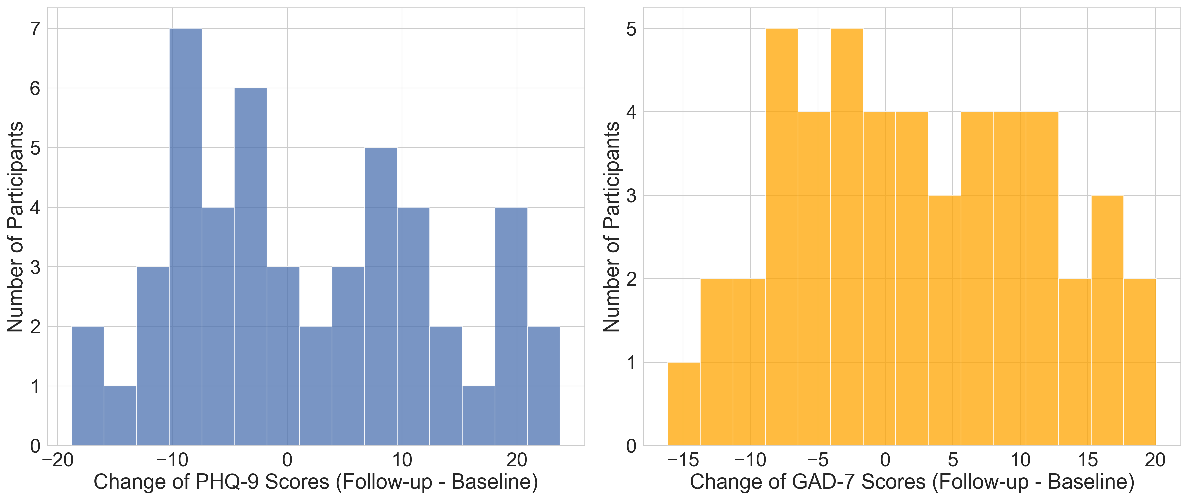
The distributions of the changes in PHQ-9 depression and GAD-7 anxiety scores before and after the lockdown. The PHQ-9 scores are shown on the left, and the GAD-7 scores are shown on the right. GAD-7: General Anxiety Disorder-7; PHQ-9: Patient Health Questionnaire-9.

Figure 4 shows the baseline (collected on January 1, 2020) and follow-up postlockdown (collected on May 31, 2020) distributions of depression and anxiety scores in our sample student population. The PHQ-9 scores are shown on the left, ranging from 0 to 27. The GAD-7 scores are shown on the right, ranging from 0 to 21. Each dot represents a participant. The x-axis represents the baseline score in January, and the y-axis represents the follow-up score during the lockdown in May. Figure 5 shows the distributions of the change in PHQ-9 depression and GAD-7 anxiety scores before and after the lockdown. Again, the PHQ-9 scores are shown on the left, and the GAD-7 scores are shown on the right. The changes were calculated as the follow-up scores in May subtracted by the baseline scores in January. Putting the pandemic into context, the deterioration in anxiety or depression levels may have been triggered by the fear of getting infected, loss of jobs, the death of family members or friends, and many other negative impacts from COVID-19. Particularly for college students, other major reasons may be the pressure of online learning, loss of financial aids, and living alone. In contrast, students that underwent quarantines with their families safely may not have shown signals of deteriorating anxiety or depression, compared to the high stress levels during normal school days.

Of the 49 participants, 61% (n=30) of the them were female, 35% (n=17) were male, and 4% (n=2) reported nonbinary genders. First-, second-, third-, and forth-year students occupied 22% (n=11), 41% (n=20), 31% (n=15), and 6% (n=3) of the whole cohort, respectively. A total of 80% (n=39) of the participants were US citizens, and the rest (n=10) were international students. A complete breakdown of demographics with respect to the deteriorating anxiety and depressive disorders are given in Table 1.

**Table 1:** Demographics of the study population.

Demographic	Increased PHQ-9^a^ (n=24), n (%)	Increased GAD-7^b^ (n=26), n (%)
Female	18 (75)	22 (85)
US citizen	18 (75)	20 (77)
First-year students	5 (21)	4 (15)
Second-year students	11 (46)	12 (46)
Third-year students	7 (29)	8 (31)
Fourth-year students	1 (4)	2 (8)

^a^PHQ-9: Patient Health Questionnaire-9.

^b^GAD-7: General Anxiety Disorder-7.

### Evaluation Outcomes

The two-tailed paired independent *t* tests mentioned at the beginning of the Statistical Analysis section were designed to rule out seasonal factors in online behavior changes and focus on COVID-19 before any of the main experiments. All features had *P* values less than .003. Hence, the presence of annual or seasonal factors accountable for online behavior changes was neglectable, and it was safe to carry out the following main experiment. This is consistent with one of the main conclusions of Huckins et al [5] that, when comparing the longitudinal data between different years, behaviors during COVID-19 shifted drastically.

We calculated the Pearson product-moment correlations, *r*, of online behavior shifts with deteriorations in anxiety and depression levels. We reported the correlation coefficients with *P* values and 95% CIs obtained for each of the aforementioned features.

#### Online Activity Distributions

For *online activity distributions*, we calculated the percentage changes in the volume of activities for all 24-hour bins separately. We obtained 24 measurements for each participant. We then evaluated for each hour of a day the correlation between the relative hourly activity change and the change in the PHQ-9 depression scores. Significant correlations were found for all hours between 10 PM and 6 AM of the next day, inclusively. All of them were positive correlations (*r* ranging between 0.32 and 0.75, all *P* values were less than or equal to .04). The correlations started increasing after 10 PM, reached the maximum at 3 AM, and started to decrease afterwards. This suggests that greater late-night online activity volumes after the lockdown may be a signal of deteriorating (increasing) depressive levels.

Next, we observed similar results from the analysis between the hourly activity change and the change in the GAD-7 anxiety scores. Significant correlations were found for all hours between 11 PM and 6 AM of the next day, inclusively. All of them were positive correlations (*r* ranging between 0.39 and 0.74, all *P* values were less than or equal to .006). The correlations started increasing after 11 PM, reached the maximum at 3 AM, and started to decrease afterwards. This implies that greater late-night online activity volumes after the lockdown may represent deteriorating (increasing) anxiety levels. For the detailed hourly correlations, 95% CIs, and comparisons between the deteriorating PHQ-9 and GAD-7 groups, see Table 2.

**Table 2:** The correlation coefficients between the change in the volume of online activities of each hour in a day and deteriorating PHQ-9 and GAD-7 scores.

Hour	Deteriorating PHQ-9^a^		Deteriorating GAD-7^b^		
	Correlation coefficient, *r* (95% CI)	*P* value		Correlation coefficient, *r* (95% CI)	*P* value
12 AM	*0.54*^c^ (0.38 to 0.72)	*<.001*		*0.45* (0.20 to 0.65)	*.001*
1 AM	*0.64* (0.45 to 0.81)	*<.001*		*0.52* (0.28 to 0.70)	*<.001*
2 AM	*0.66* (0.47 to 0.85)	*<.001*		*0.60* (0.39 to 0.75)	*<.001*
3 AM	*0.75* (0.59 to 0.94)	*<.001*		*0.74* (0.58 to 0.92)	*<.001*
4 AM	*0.63* (0.43 to 0.80)	*<.001*		*0.63* (0.43 to 0.77)	*<.001*
5 AM	*0.45* (0.29 to 0.65)	*.001*		*0.58* (0.35 to 0.74)	*<.001*
6 AM	*0.34* (0.21 to 0.54)	*.02*		*0.39* (0.12 to 0.60)	*.006*
7 AM	0.27 (0.01 to 0.54)	.06		0.24 (–0.05 to 0.48)	.10
8 AM	–0.26 (–0.50 to 0.02)	.07		–0.28 (–0.52 to 0.00)	.05
9 AM	–0.28 (–0.52 to 0.00)	.05		–0.26 (–0.50 to 0.02)	.07
10 AM	–0.27 (–0.51 to 0.01)	.06		–0.21 (–0.46 to 0.07)	.05
11 AM	–0.22 (–0.47 to 0.07)	.13		–0.25 (–0.49 to 0.03)	.08
12 PM	–0.19 (–0.44 to 0.09)	.19		–0.20 (–0.45 to 0.08)	.16
1 PM	0.23 (–0.05 to 0.48)	.10		–0.10 (–0.36 to 0.48)	.51
2 PM	0.17 (–0.12 to 0.43)	.24		0.17 (–0.12 to 0.19)	.24
3 PM	0.24 (–0.04 to 0.49)	.09		–0.22 (–0.47 to 0.06)	.12
4 PM	0.18 (–0.11 to 0.43)	.23		0.16 (–0.12 to 0.42)	.26
5 PM	0.15 (–0.14 to 0.41)	.31		0.15 (–0.14 to 0.41)	.31
6 PM	–0.16 (–0.42 to 0.13)	.27		–0.15 (–0.41 to 0.13)	.30
7 PM	–0.12 (–0.39 to 0.16)	.39		–0.24 (–0.49 to 0.04)	.10
8 PM	–0.23 (–0.47 to 0.06)	.12		–0.23 (–0.47 to 0.06)	.11
9 PM	0.25 (–0.03 to 0.50)	.08		0.24 (–0.04 to 0.49)	.09
10 PM	*0.32* (0.02 to 0.55)	*.04*		0.27 (–0.01 to 0.51)	.06
11 PM	*0.41* (0.23 to 0.62)	*.003*		*0.39* (0.13 to 0.61)	*.005*

^a^PHQ-9: Patient Health Questionnaire-9.

^b^GAD-7: General Anxiety Disorder-7.

^c^Italics indicate significant results.

#### Last Seen Activities

For last seen activities, we measured the shift in the median time of last seen activities after the lockdown. Last seen events were determined by the last activity performed before the threshold hour. First, we calculated correlation coefficients between the time shifts and changes in the PHQ-9 depression scores with different cutoff threshold hours. There was a positive correlation overall between the shift of the median time and deteriorating PHQ-9 scores. Thus, the more positive the shift (ie, the median time of the last events moved to later hours), the greater the deterioration. Specifically, for cutoff hours at 2 AM, 3 AM, 4 AM, and 5 AM, the correlations were significant (*r* ranging between 0.35 and 0.59, all *P* values were less than or equal to .01). The correlation was strongest for last seen events before 5 AM. These cutoff values were motivated by the periodic hourly online activity volumes we observed from the study population, as described in the Last Seen Activities section.

Next, we observed similar results when exploring the deterioration in GAD-7 anxiety scores. The positive correlation shows that staying up late tends to signal deteriorations in anxiety levels. For cutoff hours at 2 AM, 3 AM, 4 AM, and 5 AM, the correlations were significant (*r* ranging between 0.30 and 0.57, all *P* values were less than or equal to .03). The last seen events before 4 AM showed the most significant correlation. For the detailed hourly correlations, 95% CIs, and comparisons between the deteriorating PHQ-9 and GAD-7 groups, see Table 3.

**Table 3:** The correlation coefficients between the change in the median time of last seen events and deteriorating PHQ-9 and GAD-7 scores at different threshold hours.

Threshold	Deteriorating PHQ-9^a^			Deteriorating GAD-7^b^	
	Correlation coefficient, *r* (95% CI)	*P* value	Correlation coefficient, *r* (95% CI)		*P* value
12 AM	0.21 (–0.07 to 0.46)	.15	0.25 (–0.03 to 0.50)		.08
1 AM	0.26 (–0.02 to 0.50)	.07	0.25 (–0.03 to 0.49)		.09
2 AM	*0.35*^c^ (0.08 to 0.58)	*.01*	*0.30* (0.03 to 0.54)		*.03*
3 AM	*0.42* (0.15 to 0.65)	*.002*	*0.45* (0.18 to 0.67)		*.001*
4 AM	*0.58* (0.31 to 0.79)	*<.001*	*0.57* (0.30 to 0.78)		*<.001*
5 AM	*0.59* (0.33 to 0.80)	*<.001*	*0.56* (0.29 to 0.78)		*<.001*

^a^PHQ-9: Patient Health Questionnaire-9.

^b^GAD-7: General Anxiety Disorder-7.

^c^Italics indicate significant results.

#### SEIs

By considering Google Search and YouTube activities separately, we found different short interevent session thresholds using the method established by Halfaker et al [48]. For Google Search, the boundary between the in-session and between-session mixtures was 1 hour, which is consistent with the finding in the original paper. Thus, we set the threshold of 1 hour and considered all consecutive searches on Google within 1 hour as in the same session. By counting the numbers of adjacent searches within 1 hour before and after the lockdown, and calculating the percentage change, we did not find significant correlations between the number of short Google Search intervals and deteriorating PHQ-9 depression scores (*r*=–0.22, 95% CI –0.43 to 0.01, *P*=.06). Nor did we find significant correlations between the number of short Google Search intervals and deteriorating GAD-7 anxiety scores (*r*=–0.21, 95% CI –0.40 to 0.02, *P*=.08).

In contrast, we found that the threshold for the in-session and between-session mixtures for YouTube watching histories was 3.2 minutes, much shorter than that of Google Search. The average YouTube video interval time was 21 minutes. This threshold indicated that two adjacent videos consumed within 3.2 minutes of idle time should be considered as in the same session (ie, consecutive consumption). We then calculated the relative change of the number of such short YouTube intervals after the lockdown. We found a significant positive correlation between the increase of YouTube short intervals and deteriorating PHQ-9 scores (*r*=0.57, 95% CI 0.38 to 0.76, *P*<.001). Similarly, a significant positive correlation was found between the increase of YouTube short intervals and deteriorating GAD-7 scores (*r*=0.41, 95% CI 0.20 to 0.62, *P*=.001).

**Figure 6:**
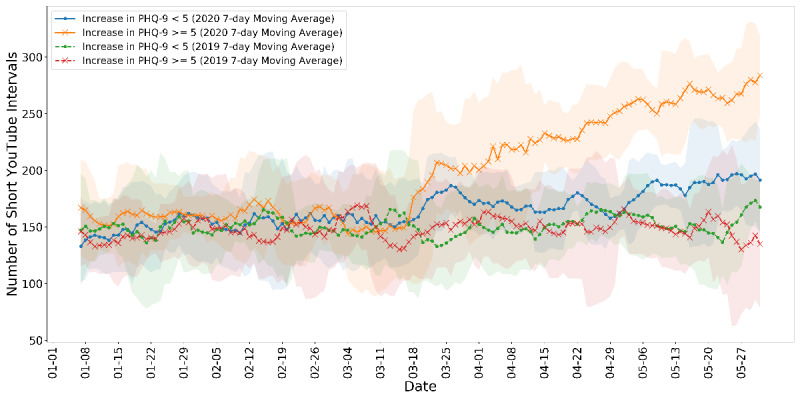
The 7-day moving average time series of the total amount of short YouTube activity intervals between groups with and without significant increases in the PHQ-9 depression scores. PHQ-9: Patient Health Questionnaire-9.

**Figure 7:**
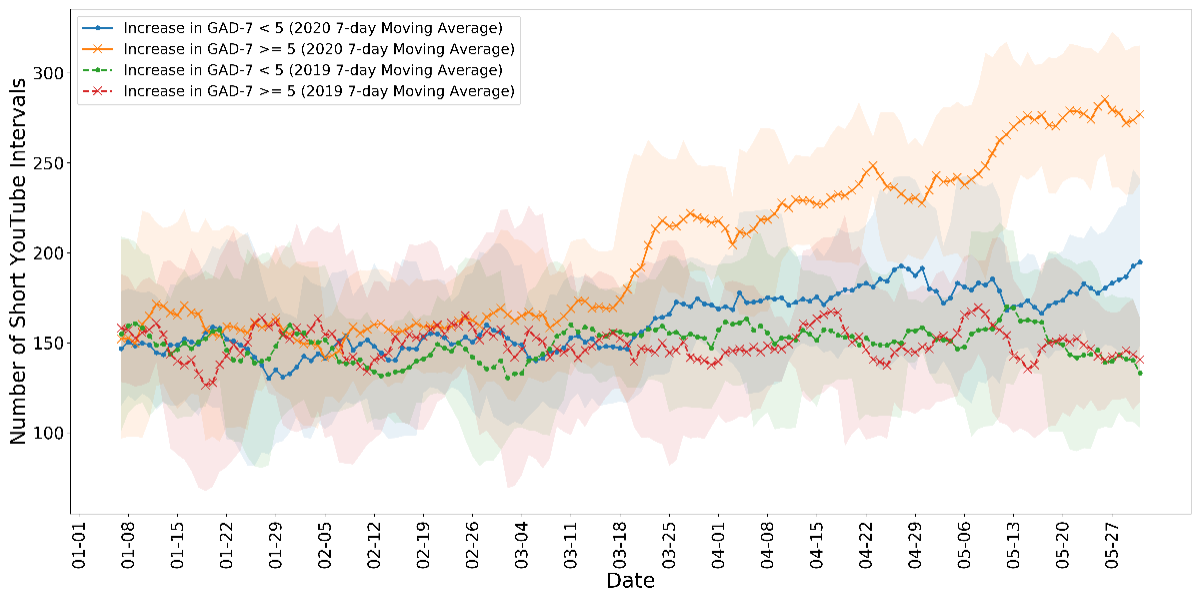
The 7-day moving average time series of the total amount of short YouTube activity intervals between groups with and without significant increases in the GAD-7 anxiety scores. GAD-7: General Anxiety Disorder-7.

In addition, we include visualizations (Figures 6 and 7) of the behavioral measurements for *SEIs* over time. As mentioned in the Measurements for Mental Health Conditions section, an increase ≥5 in PHQ-9 or GAD-7 scores is clinically alarming. Thus, we first separated the samples by groups with and without an increase ≥5 in the PHQ-9 depression scores. We then plotted the 7-day moving average total number of short YouTube event intervals (ie, consecutive video consumption) of the two groups as a function of dates. We overlayed the series with the activity data from the same time period (January 1 to May 31) in 2019 in dashed lines for contrast. As shown in Figure 6, after the lockdown in mid-March, both groups were having increasing amounts of short YouTube intervals. The participants with severe deteriorating depression outran others significantly. A similar trend was found when we separated the groups by an increase ≥5 in the GAD-7 anxiety scores, shown in Figure 7. The group with significantly deteriorated anxiety disorders tended to have higher numbers of consecutive YouTube consumption. The shaded area represents 1 SD. Most importantly, these patterns were stabilized throughout the time after the lockdown, reflecting a meaningful behavioral shift instead of mere acute or temporal observations. No such phenomenon was observed in 2019 for any group, and we further argue the fact that seasonal factors are accountable for the behavioral difference.

#### LIWC Attributes

As the contexts and linguistic properties of Google Search and YouTube may be different, we extracted the LIWC features from them separately. For Google Search, we found that search queries in the *work*, *money*, and *death* categories under the *personal concerns* dimension showed significant positive correlation with deteriorating PHQ-9 depression scores (*r* ranging between 0.33 and 0.59, all *P* values less than or equal to .02). Similar results were found for deteriorating GAD-7 anxiety scores (*r* ranging between 0.41 and 0.51, all *P* values were less than or equal to .003).

Moreover, for both depression (*r*=0.31, 95% CI 0.08 to 0.49, *P*=.03) and anxiety (*r*=0.34, 95% CI 0.10 to 0.52, *P*=.02) deteriorations, significant correlations were found in the *authentic* scores of the search queries under the *summary variable*. The *authentic* scores were developed by Newman et al [57] to provide a continuous scale for measuring how honest and genuine a given piece of text is. The higher the score, the more authentic the language. It was shown that when individuals address themselves in an authentic manner, they tend to be personal and vulnerable. For instance, a randomly chosen participant searched: “What not to do during at-home exams under proctor surveillance?” For the detailed correlations coefficients and 95% CIs, see Table 4.

**Table 4:** The significant LIWC features from Google Search histories and their correlation coefficients with deteriorating PHQ-9 and GAD-7 scores.

LIWC^a^ attributes		Deteriorating PHQ-9^b^			Deteriorating GAD-7^c^	
		*r* (95% CI)	*P* value	*r* (95% CI)		*P* value
**Personal concerns**						
	Work	*0.33*^d^ (0.09-0.53)	*.02*	*0.43* (0.22-0.60)		*.002*
	Money	*0.47* (0.28-0.60)	*.001*	*0.51* (0.33-0.63)		*<.001*
	Death	*0.59* (0.42-0.71)	*<.001*	*0.41* (0.21-0.56)		*.003*
**Summary variable**						
	Authentic	*0.31* (0.08-0.49)	*.03*	*0.34* (0.10-0.52)		*.02*

^a^LIWC: Linguistic Inquiry and Word Count.

^b^PHQ-9: Patient Health Questionnaire-9.

^c^GAD-7: General Anxiety Disorder-7.

^d^Italics indicate significant results.

For YouTube histories, we found that videos containing *anxiety* and *sadness* keywords under the *negative emotion* dimension showed significant positive correlation with deteriorating PHQ-9 depression scores (*r* ranging between 0.50 and 0.52, all *P* values less than or equal to .001). Similar results were found for deteriorating GAD-7 anxiety scores (*r* ranging between 0.55 and 0.57, all *P* values less than or equal to .001). In addition, the *friends* keywords from the *social words* dimension showed a significant positive correlation with worsened GAD-7 (*r*=0.41, 95% CI 0.21 to 0.56, *P*=.003) but not PHQ-9 (*r*=0.27, 95% CI 0.02 to 0.47, *P*=.06).

Moreover, for both depression (*r*=–0.37, 95% CI –0.16 to –0.52, *P*=.009) and anxiety (*r*=–0.47, 95% CI –0.28 to –0.60, *P*=.001) deteriorations, significant negative correlations were found in the *emotional tone* scores of the videos, under the *summary variable*. The *emotional tone* scores were developed by Cohn et al [58] to provide a continuous scale for measuring the positivity of a given piece of text and vice versa. The higher the score, the more positive the text. For the detailed correlation coefficients and 95% CIs, see Table 5.

**Table 5:** The significant LIWC features from YouTube videos and their correlation coefficients with deteriorating PHQ-9 and GAD-7 scores.

LIWC^a^ attributes		Deteriorating PHQ-9^b^		Deteriorating GAD-7^c^	
		*r* (95% CI)	*P* value	*r* (95 CI%)	*P* value
**Negative emotion**					
	Anxiety	*0.52*^d^ (0.35 to 0.64)	*<.001*	*0.57* (0.42 to 0.66)	*<.001*
	Sadness	*0.50* (0.31 to 0.63)	*<.001*	*0.55* (0.38 to 0.67)	*<.001*
**Social words**					
	Friends	*0.42* (0.22 to 0.57)	*.002*	0.27 (0.02 to 0.47)	.06
**Summary variable**					
	Emotional tone	–*0.37* (–0.16 to –0.52)	*.009*	–*0.47* (–0.28 to –0.60)	*.001*

^a^LIWC: Linguistic Inquiry and Word Count.

^b^PHQ-9: Patient Health Questionnaire-9.

^c^GAD-7: General Anxiety Disorder-7.

^d^Italics indicate significant results.

#### Google Search and YouTube Categories

The *adult* category consists of explicit browser histories of porn-related content and visiting porn sites (identified via the URL). On one hand, the percentage change of the *adult* content showed a significant positive correlation with deteriorating depression levels (*r*=0.56, 95% CI 0.35 to 0.77, *P*<.001). On the other hand, the percentage change of the *adult* content did not show a significant correlation with deteriorating anxiety levels (*r*=0.29, 95% CI 0.02 to 0.56, *P*=.08). The *news* content did not show any significant correlation with deteriorating depression (*r*=0.25, 95% CI 0.04 to 0.46, *P*=.13) nor anxiety (*r*=0.14, 95% CI –0.01 to 0.29, *P*=.21).

### Predictive Modeling

In this section, we probe the feasibility of using common machine learning models to predict the change (eg, deterioration) in PHQ-9 and GAD-7 scores. We framed the task as a supervised regression problem and treated the aforementioned behavioral changes as feature inputs to the model. The goal was to predict the change in the PHQ-9 or GAD-7 scores. Given our small sample size, we evaluated the model performance by multiple (N=49) leave-one-out train (n=48) and test (n=1) splits. For each of the splits, we tuned model hyperparameters with another complete leave-one-out cross-validation on the 48 training samples.

### Feature Vectors

We developed the feature vector by concatenating some of the significant behavioral shifts (scalars) previously mentioned. Nonetheless, given the small sample size, it was reasonable to avoid high-dimensional feature vectors. Thus, we first separated the aforementioned 5 behavioral changes into 2 groups, namely, the *temporal* and *semantic* features. Specifically, we picked the *online activity distributions* at 2 AM, 3 AM, and 4 AM; the *last seen activities* before 4 AM and 5 AM; and the *short YouTube intervals* for the *temporal* feature vector, and hence, it had a dimensionality of 6. We then concatenated the 4 significant LIWC categories for Google Search, the 4 significant LIWC categories for YouTube, and the *adult* Google Search content as the *semantic* feature vector. The dimensionality was 9.

### Regression Models

**Figure 8:**
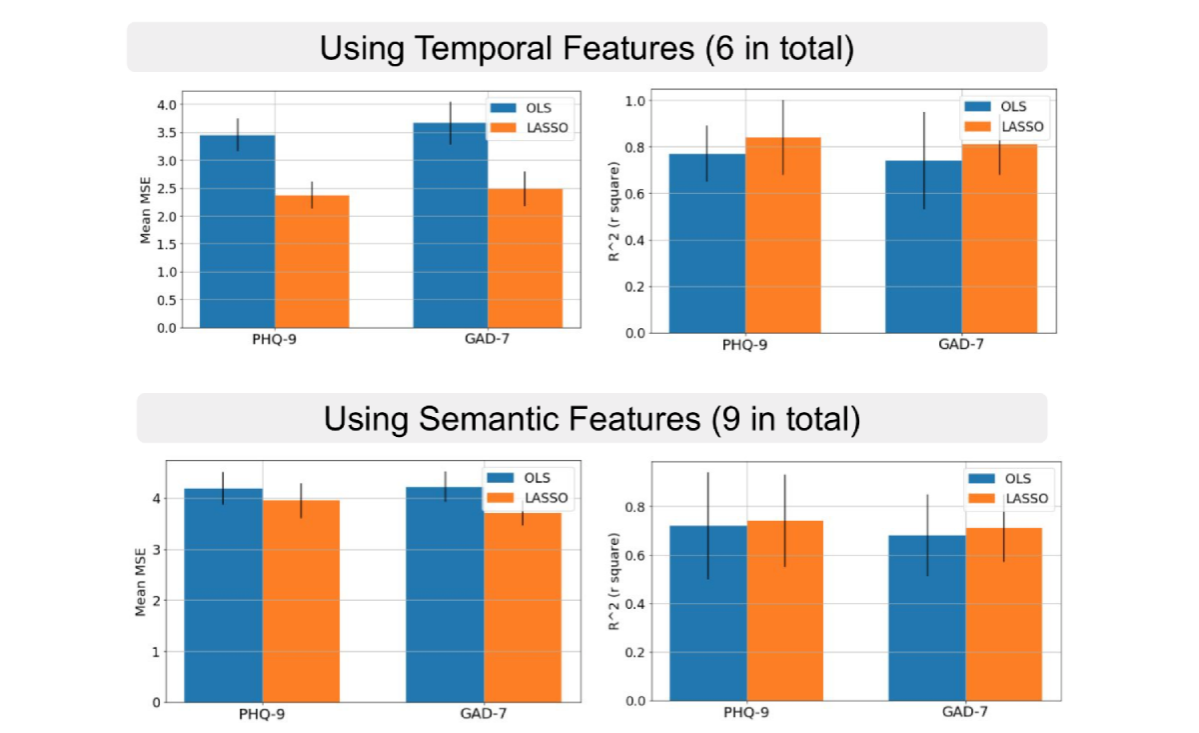
The performances of the two regression models averaged across 49 leave-one-out splits. GAD-7: General Anxiety Disorder-7; LASSO: lasso regression; MSE: mean squared error; OLS: ordinary least square regression; PHQ-9: Patient Health Questionnaire-9.

We experimented with two of the most common linear models: ordinary least square regression and lasso regression. In Figure 8, we present the performances of the regression models. We reported the mean squared error (MSE) and the average coefficient of determination (*R*^2^). The mean and SDs were calculated from the 49 leave-one-out splits. Overall, *temporal* features performed better than *semantic* ones, regardless of predicting the change in PHQ-9 or GAD-7. The best average performance in predicting the change of PHQ-9 was achieved by the *temporal* features (MSE=2.37, *R*^2^=0.84). The best average performance in predicting the change of GAD-7 was also achieved by the *temporal* features (MSE=2.48, *R*^2^=0.81).

### Model Examination

We examined the coefficient weights from the linear models for PHQ-9 and GAD-7 predictions. Since lasso regression performed the best in all cases, we looked at the weights from the fitted lasso models. For the 6 temporal features, as shown in Table 6, *online activity distributions* at 4 AM had the most significant importance in both prediction tasks. For the 9 semantic features, as shown in Table 7, keywords related to the *anxiety* dimension in the LIWC from YouTube histories had the most significant importance. Moreover, as the aforementioned statistical tests have shown that neither the *friends* dimension in LIWC nor the *adult* Google Search category were significant for deteriorating GAD-7, the lasso regression indeed assigned zero weights to these two features. We used an alpha value of .0001 for the regularization term in the lasso model.

**Table 6:** The coefficients of 6 temporal features in lasso regression.

Temporal features		Coefficient in PHQ-9^a^ prediction	Coefficient in GAD-7^b^ prediction
**Online activity distributions**			
	2 AM	0.08	0.11
	3 AM	0.34	0.28
	4 AM	0.68	0.71
**Last seen activities**			
	4 AM	0.21	0.03
	5 AM	0.64	0.54
Short YouTube intervals		0.00	0.02

^a^PHQ-9: Patient Health Questionnaire-9.

^b^GAD-7: General Anxiety Disorder-7.

**Table 7:** The coefficients of 9 semantic features in lasso regression.

Semantic features			Coefficient in PHQ-9^a^ prediction		Coefficient in GAD-7^b^ prediction
**LIWC^c^**					
	Anxiety	0.63		0.52	
	Sadness	0.48		0.47	
	Friends	0.02		0.00	
	Emotional tone	–0.23		–0.19	
	Work	0.04		0.13	
	Money	0.18		0.26	
	Death	0.01		0.07	
	Authentic	0.02		0.15	
**Google Search categories**					
	Adult	0.05		0.00	

^a^PHQ-9: Patient Health Questionnaire-9.

^b^GAD-7: General Anxiety Disorder-7.

^c^LIWC: Linguistic Inquiry and Word Count.

## Discussion

### Principal Results

In this study, we collected longitudinal individual-level Google Search and YouTube data from college students, and we measured their anxiety (GAD-7) and depression (PHQ-9) levels before and after the outbreak of COVID-19. We then developed explainable features from Google Search and YouTube logs and quantified various online behavior shifts of the participants during the pandemic. We also calculated the change in mental health conditions for all participants. Our experiment examined the correlations of online behavior features with deteriorating anxiety and depression levels. We finally demonstrated the feasibility of building simple predictive machine learning models with the proposed behavioral signals. To the best of our knowledge, we are the first to conduct observational studies on how anxiety and depression problems and Google Search and YouTube use of college students are related during COVID-19.

Our results showed that online behavior changes have significant correlations with worsened depression and anxiety profiles during the pandemic. The features we developed based on online activities were all explainable and preserved certain levels of interpretability. For example, the *SEIs* and *online activity distributions* measured the consecutive use and hourly activity volumes of Google Search and YouTube, which were inspired by previous studies on excessive YouTube use [26], internet addictions [59], and positive associations with social anxiety among college students [27]. Our results indicated that individuals with increasing anxiety or depressive disorders during the pandemic tended to have long use sessions (multiple consecutive activities with short time intervals) when engaging with Google Search and YouTube.

Moreover, the increasing activities during late night hours in *online activity distributions* and the positive shifts of medians of *last seen events* corresponded with previous studies in sleep deprivation and subsequent positive correlations with mental health deteriorations [60,61]. Our results demonstrated that individuals with worsened anxiety or depressive symptoms during the pandemic were indeed likely to stay up late and engage more online. The aforementioned three features captured the temporal aspects of user online behaviors, and they generated the best performance in the regression tasks.

Additionally, our analysis found that the amount of porn consumption had significant correlations with deteriorating depression, which adheres to previous findings that people with depression and loneliness are likely to consume excessive pornography [62,63]. For the LIWC features, participants with significant increases in anxiety watched more videos with *anxiety*, *sadness*, and negative tone, and previous research has shown that negative YouTube videos tended to receive more attention from vulnerable individuals [64]. They also consumed more videos related to social activities and *friends* keywords. The *friends* keywords did not show any significance in the depression analysis. This is consistent with studies on patterns of social withdrawal and depression [42,65,66], and social interactions and isolations have been recognized by Leigh-Hunt et al [67] as one of the priorities in mental illness prevention, especially during COVID-19 [30]. For Google Search, both the participants with significant increases in anxiety and depression searched more content related to *work*, *money*, and *death*, focusing on real-life practices. None of the emotional dimensions were significant in Google Search logs. Instead, LIWC considered the search queries from depressed and anxious individuals more honest and vulnerable (eg, asking for help) after the lockdown, given the *authentic* score. Although prior research has shown that individuals living with depression tend to use more first-person languages [68], we did not observe any similar pattern. This is probably due to the fact that search queries are more succinct, imperative, and functional, which leaves less necessity for personal references. These attributes captured the semantic aspect of user online behaviors. The prevalence of personal affair, social activity, and negative keywords as well as porn consumption has shown statistically significant correlations.

Many researchers have reported that there has been a significant boost in health- and news-related topics at the population level in various online platforms during COVID-19. This is partly due to additional measures taken by individuals, various stakeholders, and agencies with regard to preventive measures [11,36,37], daily statistics [10,12,13], and health care information and misinformation [35,37,38]. However, unlike many, our investigation was carried out considering individual-level Google Search and YouTube engagement logs, and our analysis did not reveal any significant spikes in the *news* and *health and illness* categories among individuals with deteriorating anxiety and depression during the pandemic. One possible explanation for such observation could be due to the target population (college students) of our study, who may prefer to follow news from other popular platforms such as social media.

Finally, COVID-19 has forced us to alter daily lifestyles. The world was not ready for such a viral outbreak. Since there is no cure for COVID-19, it, or an even more deadly viral disease, may resurface at different capacities in the near future. Society may be forced to rely on technologies even more and employ remote learning, working, and socializing for a longer period of time. It is important that we learn from our experience of living through the initial COVID-19 outbreak and take necessary measures to uncover the changes in online behaviors, investigating how that can be leveraged to understand and monitor various mental health conditions of individuals in the least invasive manner. Furthermore, we hope our work paves the path for technology stakeholders to consider incorporating various mental health assessment monitoring systems using user engagements, following users’ consent in a privacy-preserving manner. They can periodically share the mental health monitoring assessment report with users based on their online activities and education, and inform users about their current mental health. This can eventually encourage individuals to acknowledge the importance of mental health and take better care of themselves.

### Limitations

First, although most of the online behavioral features we developed showed significant correlations, our study cohort only represented a small portion of the whole population with mental health difficulties. Therefore, further studies are required to investigate if the significant behavioral changes still hold among more general communities not limited to college students. It is possible that the relationship of worsened anxiety and depression with online activities on Google Search and YouTube of our college population is different from that of other populations whose education and social backgrounds may vary. There may also be differences in mental health for substudent populations, such as those living in harmful environments and those depending on financial aid, all of whom may experience more physical and economic crises during COVID-19. Nonetheless, we argue that the explainable features we constructed, such as late-night activities, continuous use, inactivity, pornography, and certain keywords, can remain behaviorally representative and be applied universally across experiments exploring the relationship of anxiety and depression with online activities during the pandemic.

Second, in this study, we explored the relationship between user online behaviors and the fluctuations in anxiety and depression conditions during COVID-19. Any causal relationship between online behavior and mental disorders is beyond the scope of this study. As one can readily imagine, online behavioral changes could both contribute to or be caused by deteriorating anxiety or depressive disorders.

Third, we acknowledge that it may be impossible to obtain data without noise because one may seldom, or even never, search on Google or watch YouTube videos. Such concealed information makes it impossible for the proposed model to flag alarming symptoms that are reflected in the PHQ-9 and GAD-7 questionnaires.

Fourth, though we included preliminary demographic information as covariates, there remains the possibility of other confounding factors. In fact, both the shifts in online behaviors and deteriorating mental health profiles may be due to common factors such as living conditions, financial difficulties, and other health problems during the pandemic. There was also no causal direction implied between COVID-19 and online behavior changes, which was introduced in the first paragraph of the Statistical Analysis section as a precaution before the main experiments.

### Ethical and Privacy Concerns

Despite being a pilot study, our results indicate that it is possible to build an anxiety and depression surveillance system based on passively collected private Google data histories during COVID-19. Such noninvasive systems shall be subject to rigorous data security and anonymity checks. Necessary measures need to be in place to ensure personal safety and privacy concerns when collecting sensitive and proprietary data such as Google Search logs and YouTube histories. Even in pilot studies, participants should have full rights over their data; they may choose to opt out of the study at any stage and remove any data shared in the system.

Moreover, anonymity and systematic bias elimination should be enforced. As an automatic medical screening system based on pervasive data, it has been shown that such frameworks are prone to implicit machine learning bias during data collection or training phases [69-71]. Black-box methods should be avoided, as they are known to be vulnerable to adversarial attacks and produce unexplainable distributional representations [72,73]. Anonymizing data and obscuring identity information should be the first step in data debiasing.

In the end, to what extent should caregivers trust a clinical decision made by machines remains an open question. We believe that possible pervasive computing frameworks shall play the role of a smart assistant, at most, to the care providers. Any final intervention or help delivery decision should be made by health care professionals who understand both the mental health problems and the limitations of automatic detection systems in clinical settings.

## Abbreviations

API: application programming interface
GAD-7: General Anxiety Disorder-7
LIWC: Linguistic Inquiry and Word Count
MSE: mean squared error
NLP: natural language processing
PHQ-9: Patient Health Questionnaire-9
S: slope
SEI: short event interval
